# Six-Month Outcome in Bipolar Spectrum Alcoholics Treated with Acamprosate after Detoxification: A Retrospective Study

**DOI:** 10.3390/ijerph111212983

**Published:** 2014-12-12

**Authors:** Angelo Giovanni Icro Maremmani, Silvia Bacciardi, Luca Rovai, Fabio Rugani, Enrico Massimetti, Denise Gazzarrini, Liliana Dell’Osso, Icro Maremmani

**Affiliations:** 1 Vincent P. Dole Dual Diagnosis Unit, Department of Neurosciences, Santa Chiara University Hospital, University of Pisa, Pisa 56100, Italy; E-Mails: angelogimaremmani@gmail.com (A.G.I.M.); s.baciard@gmail.com (S.B.); lucarovai@yahoo.com (L.R.); fabruga@gmail.com (F.R.); e.massimetti@yahoo.it (E.M.); denise.gazzarrini@hotmail.it (D.G.); 2 Association for the Application of Neuroscientific Knowledge to Social Aims (AU-CNS), Pietrasanta, Lucca 55045, Italy; 3 Section of Psychiatry, Department of Experimental and Clinical Medicine, University of Pisa, Pisa 56100, Italy; E-Mail: liliana.dellosso@med.unipi.it; 4 G. De Lisio Institute of Behavioural Sciences, Pisa 56100, Italy

**Keywords:** acamprosate, depression, bipolarity, long-term outcome, glutamate

## Abstract

*Background*: Glutamate system is modified by ethanol and contributes both to the euphoric and the dysphoric consequences of intoxication, but there is now growing evidence that the glutamatergic system also plays a central role in the neurobiology and treatment of mood disorders, including major depressive disorders and bipolar disorders. We speculate that, using acamprosate, patients with bipolar depression (BIP-A) can take advantage of the anti-glutamate effect of acamprosate to “survive” in treatment longer than peers suffering from non-bipolar depression (NBIP-A) after detoxification. *Method*: We retrospectively evaluated the efficacy of a long-term (six-month) acamprosate treatment, after alcohol detoxification, in 41 patients (19 males and 22 females), who could be classified as depressed alcoholics, while taking into account the presence/absence of bipolarity. *Results*: During the period of observation most NBIP-A patients relapsed, whereas a majority of BIP-A patients were still in treatment at the end of their period of observation. The cumulative proportion of ‘surviving’ patients was significantly higher in BIP-A patients, but this finding was not related to gender or to other demographic or clinically investigated characteristics. The treatment time effect was significant in both subgroups. The treatment time-group effect was significant (and significantly better) for bipolar patients on account of changes in the severity of their illness. *Limitations*: Retrospective methodology and the lack of DSM criteria in diagnosing bipolarity. *Conclusions*: Bipolarity seems to be correlated with the efficacy of acamprosate treatment in inducing patients to refrain from alcohol use after detoxification (while avoiding relapses) in depressed alcoholics. Placebo-controlled clinical trials are now warranted to check the validity of this hypothesis.

## 1. Introduction

Glutamate is the primary excitatory neurotransmitter in the brain. Glutamatergic systems are targets for the actions of ethanol via its antagonism towards the N-methyl-D-aspartate (NMDA) subtype of the glutamate receptor and other mechanisms. The modulation of glutamatergic function by ethanol contributes both to the euphoric and the dysphoric consequences of ethanol intoxication [1,2]. In the last few decades, glutamate has been studied as a neurotransmitter that could play a central role in the processes underlying the development and maintenance of addiction. These processes include reinforcement, sensitization, habit learning and reinforcement learning, context conditioning, craving and relapse [3]; this supports the idea that alcoholism could be considered another member of the expanding family of glutamate-related neuropsychiatric disorders. As mentioned above, ethanol interferes with glutamatergic neurotransmission, one acute effect being the inhibition of NMDA receptors. Prolonged inhibition of these receptors by ethanol results in the development of supersensitivity; acute removal of ethanol causes a marked increase in the activity of postsynaptic neurons, such as those in the noradrenergic system, and, to an extreme degree, glutamate-induced excitotoxicity [4].

Acting as the major excitatory neurotransmitter in the cerebral cortex, glutamate is thought to play a role in major mental disorders, including major depressive disorders and bipolar disorders [5,6,7,8,9]. Several studies report an increase in the level of glutamate during mania. The overexpression of the glutamatergic system in brain regions such as the dorsolateral prefrontal cortex (with special reference to left-sided structural and metabolic abnormalities) during manic phases could function as a link between glutamate and excitement [10,11]. In mania, there is a probable correlation between excessive glutamatergic neurotransmission driven by neuronal activity, and phenomena such as racing thoughts, distractibility, irritability and insomnia [12]. In line with these observations, in non-manic people the glutamine/glutamate ratio, an index that successfully captures the brain glutamatergic activity, has been found to be lower [13].

Although the relationship between substance abuse and bipolar disorder is complex and not yet fully understood, the idea that they could share mechanisms of self-renewal and positive mutual reinforcement has already been raised [14,15,16,17,18,19,20]. A common background to these two psychopathological entities seems to be impulsivity which, together with excitement, appears to be the triggering and reinforcing factor for the continuing use of drugs in bipolar patients [21,22]. Risk-taking behaviours during hypomanic states are broadly recognized as being an integral part of bipolar illness [23]. Given the fact that alcoholism is more frequent in bipolar than unipolar patients, heavy drinking is fairly common when patients are hypomanic [23,24]. Moreover, in bipolar patients, familial diathesis for mania is significantly associated with alcohol and other drugs of abuse.

We speculate that, by using acamprosate, patients with bipolar depression can take advantage of the anti-glutamate effect of acamprosate to stay in treatment for a longer time than peers with non-bipolar depression after detoxification. This hypothesis implies that acamprosate would be able to reduce the drive to drink with the outcome that their time to relapse would be longer that of peers without bipolar depression after detoxification.

In our clinical practice, we started considering bipolarity in all alcoholic depressed patients treated with acamprosate. The main aim of the present study was to retrospectively evaluate the efficacy of a long-term (six-month) acamprosate treatment in depressed alcoholics, dividing those showing bipolarity from those without bipolarity, after alcohol detoxification, with reference to the following parameters:

- Total Alcohol Consumption (TAC).
- Time to relapse, measured in days. We consider a *“relapse”* to have taken place if a male patient takes more than 2 drinks on one day or if a female patient takes more than 1 drinks on one day (expressed in terms of standard US drinks; one standard U.S. drink = 14 g absolute alcohol) [25]; we do not consider patients as having relapsed if there has only been a “slip”, which can be defined as an occasion when a male patient takes no more than 2 drinks on one day or when a female patient takes 1 drink on one day. With this kind of outcome, of course, we did not consider that there had been a “relapse into heavy drinking days” (relapse-HDD), which happens when a male patient takes 5 or more drinks on a single day or a female patient takes 4 or more drinks on a single day [25].
- Clinical Global index (CGI).
- Global functioning assessment (GAF).
- Time in days with TAC = 0.

The secondary aim was to evaluate the correlation between demographic, clinical variables and retention in treatment.

## 2. Experimental Section

### 2.1. Sample

We retrospectively considered all consecutive depressed chronic alcoholics in treatment with acamprosate at the Dual Diagnosis Unit of the Department of Psychiatry of the University of Pisa, Italy, during a three-year period, 2010–2013. The inclusion criteria were:

- Diagnosis of alcohol dependence according to DSM-IV-R criteria [26].
- Diagnosis of major depressive episode according to DSM-IV-R criteria.
- Presence of multiple psychosocial or environmental problems within the previous two years.
- Patients consuming over 5 units of alcohol per day at treatment entry.
- Patients living with their families.
- Having successfully accomplished 7 days of a detoxification program with sodium oxybate.
- Having successfully recovered from a depressive state after 3 weeks, whether using or not using serotoninergic agents (if used, serotoninergic agents were prescribed for a short period of time, at most 3 weeks).
- Patients not treated with antiepileptics.

Exclusion criteria were:

- Serious liver disorders and chronic diseases.

The sample consisted of 41 patients, 19 (46.3%) of them males and 22 (53.7) females, average age 40.88 ± 11.5 (min 17, max 66). Patients were mostly involved in a stable sexual relationship (n = 25; 65.8%), and were currently blue collar (n = 26; 65.0%), with a low educational level (n = 24; 64.9%), without welfare benefits (n = 34; 85.0%), with adequate income (n = 35; 92.1%), but experiencing difficulties as to social adjustment, and living in a family (n = 33; 80.5%). According to the Hypomania Check-List (HCL) cut-off, 22 patients showed a depressive episode in the absence of bipolar spectrum (NBIP-A patients). 11 (50.0%) were males, age ranged between 26 and 38 years (average age 43.50 ± 10.9). 19 were affected by bipolar depression (HCL score ≤ 14) (BIP-A patients). 8 (42.6%) were males, age ranged between 17 and 66 years (average age 37.84 ± 11.8).

### 2.2. Instruments

The following instruments were used to collect data on the variables to be studied:

- Demographic data (at the beginning of treatment). We considered gender (males, females), age (≤40 years, ≥40), education (≤8 years, ≥8 years), marital status (single, married), job status (white collars, blue collars, unemployed), income (poor, adequate).
- Bipolar spectrum. To divide our sample into patients with and without bipolar spectrum, we used the Hypomania Check-List (HCL) compiled by Angst [27,28]. This is a checklist of 32 possible symptoms of hypomania that are rated “yes” (present or typical of me) or “no” (not present or not typical) by the subject. The cut-off for the discrimination between unipolar and bipolar patients is fixed at a score of 14/32.
- Alcohol intake (according to scheduled visits). This was evaluated in terms of units of alcohol. The easiest way to calculate this is to count the number of glasses of alcoholic drinks consumed daily, expressed as standard US drinks (one standard U.S. drink = 14 g absolute alcohol) [25].
- TAC = 0 (according to scheduled visits). This parameter was assessed through self-evaluation, and confirmed by family observer evaluation. One or two members of the family were responsible for detecting the intake of the medications and patient alcohol intake.
- Clinical Global Impressions (CGI) (monthly). Severity of illness, global improvement and efficacy index were evaluated by CGI [29]. Clinical Global Impressions (CGI) is an index that consists of three global scales (items). Two of the items—Severity of Illness and Global Improvement are rated on a 7-point scale; while the third, Efficacy Index, requires an assessment of the interaction of therapeutic effectiveness with adverse reactions.
- Social adjustment (monthly). This was evaluated by means of the Global Assessment of Functioning (GAF) [26]. The GAF reports the clinician’s judgment on the individual’s overall level of functioning classified as variation from the minimum (0) to the maximum (100) level.

A researcher who had not been informed about variations in subjects’ alcohol intake administered the CGI and GAF. There was a preference for the researcher not to be informed because CGI and GAF scales are to be rated exclusively with respect to psychological, psychopathological, social and occupational functioning.

### 2.3. Data Analysis

Patients were assessed monthly by the use of CGI and GAF. Patients who stayed in treatment were assessed at the end of treatment. When patients had negative outcomes, those who gave up were assessed at the time of treatment interruption, this being counted as their last regular assessment, rather than the previous month’s.

Depressed patients with and without bipolarity were compared for demographic and clinical aspects by means of the chi-square test for categorical variables, and Student’s t test for continuous variables. Retention in treatment was analysed by means of the survival analysis and Wilcoxon statistics to allow the survival curves to be compared. For the purposes of this analysis, the term “terminal event” refers to patients who left the treatment after a “relapse”, while “withdrawing during interval” refers to patients who are still in treatment at the end-point, or have decided to leave treatment for reasons unrelated to the treatment itself (e.g., patients moving to other towns or cities). In other words, we consider 2 kinds of positive outcome: the first when a patient left the treatment programme with a successful result (whether because abstinent or despite a “slip”) or was referred, in the same condition, to other programmes; the second when a patient was still in treatment, at the end-point, either as an abstainer, or despite a “slip”. We consider a negative outcome to have happened if a patient has “relapsed”.

Regarding clinical global impressions and social adjustment outcomes, univariate and multivariate statistical procedures have been used for cross-sectional evaluation and repeated analysis of variance for longitudinal evaluations.

The statistical tests were considered significant at the level of *p* < 0.05. We made use of the statistical routines of SPSS 20.0 (IBM Corp., Armonk, NY, USA).

## 3. Results and Discussion

### 3.1. Baseline Evaluation (at the Beginning of the Treatment)

On the basis of the demographic information that had been collected, no differences were observed, at the beginning of treatment, between BIP-A and NBIP-A patients. TAC (alcohol units/day) did not differ between BIP-A (7.74 ± 1.6) and NBIP-A (7.73 ± 1.4) patients. No differences were observed either in the CGI severity of illness (BIP-A = 5.79 ± 0.7; NBIP-A = 5.73 ± 0.7), or the GAF scores (BIP-A = 45.00 ± 4.7; NBIP-A = 43.18 ± 5.6).

### 3.2. Relapsing and Non-Relapsing Patients

At the end of six months, 5 (22.7%) NBIP-A and 12 (63.2%) BIP-A patients were still in treatment, while 17 (77.3%) NBIP-A and 7 (36.8%) BIP-A patients had relapsed. This difference was statistically significant (chi square 6.86, df = 1, *p* = 0.009). None of the patients had been dismissed for being violent; none had given the treatment up because of side-effects; none had been imprisoned or hospitalized.

At the end of six months 8 (36.4%) NBIP-A and 9 (47.4%) BIP-A patients showed uninterrupted TAC = 0; 14 (63.6) NBIP-A and 10 (52.6%) BIP-A patients showed slips (chi-square = 0.50; df = 1; *p* = 0.476).

### 3.3. Retention in Treatment

Table 1 shows retention in treatment in our sample. The cumulative proportion of patients “surviving” at the end of the observational period was significantly higher in BIP-A patients. The female (0.29) and male (0.48) retention rates were not statistically different (Wilcoxon statistics = 2.53, df = 1, *p* = 0.111). BIP-A males showed a better retention rate (0.75) than their NBIP-A peers (0.24) (Wilcoxon statistics = 6.37, df = 1, *p* = 0.012). BIP-A females showed a better retention rate (0.16) than NBIP-A females (0.09) (Wilcoxon statistics = 4.77, df = 1, *p* = 0.029).

**Table 1:** “Survival” in treatment of depressed alcoholics with and without bipolar spectrum, treated with acamprosate after detoxification.

Start Time(Months)	NumberEnteringInterval		NumberWithdrawingduringInterval		Number ofTerminalEvents		Cumulative Proportion “Surviving” at End of Given Interval	
	Bipolar Spectrum		Bipolar Spectrum		Bipolar Spectrum		Bipolar Spectrum	
	No	Yes	No	Yes	No	Yes	No	Yes
0	22	19	0	0	1	2	0.95	0.89
1	21	17	2	0	11	0	0.43	0.89
2	8	17	0	0	3	1	0.27	0.84
3	5	16	0	1	1	1	0.21	0.79
4	4	14	1	1	1	2	0.15	0.67
5	2	11	0	0	0	1	0.15	0.61
6	2	10	2	10	0	0	0.15	0.61

Overall Comparison, Wilcoxon statistics = 10.231, df = 1, *p* = 0.001.

### 3.4. End Point Evaluation

The percentage of TAC = 0 time in days was 0.88 ± 0.1 in BIP-A and 0.89 ± 0.1 in NBIP-A patients (T = −0.28; *p* = 0.77). CGI improvement was assessed as “much improved” in BIP-A (1.79 ± 0.7) and NBIP-A (2.09 ± 1.3) patients (T = 0.93; *p* = 0.376). CGI efficacy index differed in BIP-A and NBIP-A patients (Table 2).

**Table 2:** Efficacy index in alcoholics with and without bipolar spectrum treated with acamprosate after detoxification.

CGI Efficacy Index	Depressed Alcoholics (N, %)	
	Without Bipolar Spectrumn = 22	With Bipolar Spectrumn = 19
Marked therapeutic effect - No side-effects	4 (18.2%) **^a^**	7 (36.8%) **^a^**
Marked therapeutic effect - Does not interfere with patients’ functioning	2 (9.1%) **^a^**	5 (26.3%) **^a^**
Moderate therapeutic effect - No side-effects	5 (22.7%) **^a^**	3 (15.8%) **^a^**
Moderate therapeutic effect - Does not interfere with patients’ functioning	11 (50.0%) **^a^**	4 (21.1%) **^b^**
No therapeutic effect – No side-effects	2 (9.1%) **^a^**	2 (10.5%) **^a^**

Each letter denotes a subset of categories whose column proportions do not differ significantly from each other at the 0.05 level.

BIP-A patients reported a lower CGI severity of illness (2.41 ± 1.3) than NBIP-A patients (3.21 ± 1.5). Time effect (F = 132.73, df = 1, *p* = 0.000), and time-group effect (F = 4.79, df = 1, *p* = 0.035) were significant. Time-group differences were not related to the outcome (F = 0.75, df = 1, *p* = 0.392). BIP-A patients (61.84 ± 10.1) and NBIP-A patients (62.73 ± 13.3) did not report significantly different degrees of social adjustment. Time effect was significant (F = 63.55, df = 1, *p* = 0.000). Time-group effect (F = 0.25, df = 1, *p* = 0.620) was not significant and was not related to the outcome (F = 0.88, df = 1, *p* = 0.769).

### 3.5. Correlations between Demographic, Clinical Variables and Retention in Treatment

Using Cox regression life table statistics, stepwise forward (Wald) method, (chi-square = 11.97, df = 11, *p* = 0.365), no correlation was found between retention rate and gender, age (≤40 years, >40 years), education, marital status, job, income, severity of illness, social adjustment at study entry, or complete abstinence from alcohol.

### 3.6. Medications

In this study the following were prescribed:

- Acamprosate: 1.332 and 1.998 g/die if body weight was less and more than 70 kg, respectively.
- Sodium-oxybate: 100mg/kg/die, during the first 7 days as preferred detoxification procedure.
- Antidepressant (serotoninergic): paroxetine (up to 20 mg/die), sertraline (up to 150 mg/die), citalopram (up to 10 mg/die), fluvoxamine (up to 200 mg/die).

During the period of observation most NBIP-A patients relapsed, whereas most BIP-A patients were still in treatment at the end of the period of observation. The cumulative proportion of “surviving” patients was significantly higher in BIP-A patients and showed no correlation with gender or tother demographically or clinically investigated characteristics. Treatment time effect was significant regarding CGI and GAF in both subgroups. Considering differences between the two groups, the treatment time effect was significant (significantly better) in BIP-A patients for a single parameter: CGI severity of illness. More frequently the therapeutic effect was assessed as marked in BIP-A patients and as moderate in NBIP-A patients.

At the end of our period of observation, more BIP-A patients, percentagewise, were still in treatment, whereas a higher percentage of NBIP-A patients had relapsed. In line with this observation, some studies have pointed out that bipolar alcoholics had a better outcome, as assessed by drinks per drinking day, than depressed alcoholics after a period of observation as long as 2 years [30], while others argue that these differences in outcome deriving from bipolarity are present but not statistically significant [31]. One consideration on retention in treatment pertinent to the role of dual diagnosis should be borne in mind: the higher retention percentage of BIP-A patients could be due to the better outcome achieved by dual diagnosis patients with respect to those without dual diagnosis [32]. If we focus on the length of the follow-up period, other studies highlight the usefulness of acamprosate at 6-months follow-up [33], especially in patients motivated to have a treatment goal of abstinence [34] or in patients who have benefited from forms of psychosocial supports [35,36]. In any cases, some Authors showed acamprosate treatment over 180 days to be consistently more effective than placebo in maintaining abstinence and in diminishing relapse severity [37], while others highlighted the finding that the effectiveness of acamprosate was similar to that of placebo if the administration of acamprosate was started long after detoxification [38].

Gender is an important variable in considering differences in the prevalence, risk, and clinical correlates of alcoholism in bipolar illness. While the prevalence of alcohol abuse or dependence is higher for bipolar men than bipolar women, the risk of developing alcoholism, by comparison with the general population is significantly higher for bipolar women [39,40]. Despite these distinctions, within our sample gender did not seem to influence retention in treatment. This is in line with other studies, and this finding can be considered specifically true for those dependent on alcohol [41,42,43], but it could also be extended to other substance use disorders, such as dependence on heroin [44].

On the issue of endpoint evaluations, BIP-A patients reported a lower severity of illness than NBIP-A patients. Time effect and time-group effect were both significant. Moreover, time-group differences were unrelated to the outcome. These are notable findings, especially if one considers the low degree of compliance with treatments for drug addiction and for several forms of psychiatric disorder. Looking at outcome and retention in treatment, there are studies that have stressed the fact that substance use and alcohol use disorders are associated with poor clinical outcomes in patients who have a bipolar disorder [45,46,47,48,49].

Acamprosate shows attractive features (e.g., its pharmacokinetic and safety properties), but less than thrilling results: negative trials have been reported as well as large meta-analyses that support the concept that acamprosate efficacy is limited [34,38,41,50]. One important question that arises is whether there is a specific subgroup of patients who respond particularly well to this medication. Acamprosate has proved to be most effective in acting on a hyper-glutamatergic system [51] that is also involved in the pathophysiology of several psychiatric diseases such as bipolar disorder and bipolar depression [9,52,53,54,55]. Adopting this perspective, acamprosate would be of special interest in alcohol-dependent bipolar disorder patients [56,57,58]. A randomized, placebo-controlled clinical trial of add-on acamprosate on mood stabilizing medication in 33 bipolar II or I disorder adults with alcohol dependence did not show overall differences in drinking outcomes, but acamprosate does appears to confer some clinical benefit on study completers [59]. So far, however, remarkably little research has been carried out on the concurrent trajectories of mood symptoms and alcohol use in people with co-occurring bipolar disorder and alcohol use disorders, and divergent results have been reported [60,61,62,63]. Depression has been investigated as a predictor of relapse in alcohol use. Results from a study by Priscindaro *et al.* suggest that depressive symptoms and alcohol craving both increase the proximal risks of alcohol use in individuals with co-occurring bipolar and alcohol use disorders [64]. In reviewing mood disorders as a whole, Koukopoulos and coll. suggested the need to stress the “primacy of mania” hypothesis: there is not only an intrinsic link between mania and depression, but the excitatory process of mania functions should be seen as a primary process, with depression being a secondary result [65]. The efficacy of anti-glutamate drugs in bipolar depression appears to be mediated by lowering levels of cerebral glutamate and/or glutamine, in order to prevent the exitotoxicity induced by excessive stimulation [66,67]. We can speculate that, in our patients, the antimanic effect of acamprosate, by preventing mood instability and elation, will probably reduce the risks of relapse into alcohol use that are induced by excitement [68]. Anti-glutamatergic medications appear to provide a clinically substantial antimanic, sustained mood-stabilizing effect and they seem to be useful tools in treating bipolar-depression by suppressing the excitement process that leads to mania [69].

## 4. Limitations

This is a retrospective study, so we can only put forward a suggestion about the possible correlation between acamprosate and a positive outcome in bipolar alcoholics after detoxification. We decided not to use DSM criteria to detect bipolarity, preferring HCL because of its higher sensitivity [19,70]. We chose “retention in treatment” as being the primary outcome. Subjects who “survive” in treatment are those who have dramatically decreased their alcohol intake; by contrast, patients who “die” in treatment are those who have *relapsed* rather than *slipped* back into alcohol use).

## 5. Conclusions

After detoxification from alcohol, BIP-A depressed patients stay in treatment longer than their NBIP-A depressed peers. These results highlight the fact that bipolarity seems to be significantly correlated with the outcome of acamprosate treatment, in terms of the percentage of depressed patients who refrain from alcohol use after their detoxification. Of course that placebo-controlled clinical trials are now warranted and required to check the validity of this hypothesis.
